# Self-Organized Criticality Theory and the Expansion of PD-1-Positive Effector CD4 T Cells: Search for Autoantibody-Inducing CD4 T Cells

**DOI:** 10.3389/fimmu.2013.00087

**Published:** 2013-04-10

**Authors:** Yumi Miyazaki, Ken Tsumiyama, Takashi Yamane, Mitsuhiro Ito, Shunichi Shiozawa

**Affiliations:** ^1^Department of Medicine, Kyushu University Beppu HospitalBeppu, Japan; ^2^Rheumatic Diseases Center, Konan Kakogawa HospitalKakogawa, Japan; ^3^Department of Biophysics, Graduate School of Health Sciences, Kobe UniversityKobe, Japan; ^4^Global Center of ExcellenceJapan

## Self-Organized Criticality Theory and the Cause of SLE

The cause of systemic lupus erythematosus (SLE) remains unknown (Fu et al., 2011; Perry et al., 2011). A critical question in elucidating the pathogenesis of SLE or autoimmunity would be how autoreactive clones emerge and expand in the host. According to the prevailing view of autoimmune disease, autoreactive clones may come from either clones that had escaped negative selection in the thymus or clones that have been reactivated from tolerance. However, clones that would emerge by either process would be restricted in their antigen specificity and apparently could not account for the broad T cell receptor (TCR) repertoire and the more than 100 distinct autoantibody specificities found in SLE (Shiozawa, 2012).

We have subsequently developed an alternative novel theory, called the self-organized criticality theory, which proposes that autoreactive lymphocyte clones are newly generated *via de novo* TCR revision from non-autoreactive clones at peripheral lymphoid organs (Tsumiyama et al., 2009). We identified a CD4 T cell subset that has passed through TCRα but not TCRβ revision at peripheral lymphoid organ spleen, and we named this an “autoantibody-inducing CD4 T cell” (*ai*CD4 T cell).

The term “self-organized criticality” is derived from systems engineering. The theory proposes that systemic autoimmunity or SLE, necessarily takes place when the host's immune system is overdriven by repeated exposure to antigen, reaching levels that surpass the immune system's stability-limit, i.e., self-organized criticality (Tsumiyama et al., 2009; Shiozawa, 2011, 2012). In our experiments, the novel T cells that result from such overstimulation, i.e., *ai*CD4 T cells, not only could induce B cells to generate a large variety of autoantibodies, but also promote final differentiation of CD8 T cells into cytotoxic T lymphocytes (CTL) *via* antigen cross-presentation, leading to the tissue injuries identical to those seen in SLE (Tsumiyama et al., 2009). Thus, this *ai*CD4 T cell is functionally indispensable for the pathogenesis of SLE. However, a precise characterization of these cells is still in progress, and we report here our recent findings on the distinct cell surface markers that characterize and identify *ai*CD4 T cells.

## The *ai*CD4 T Cell and PD-1-Positive Effector CD4 T Cell

**Figure 1:**
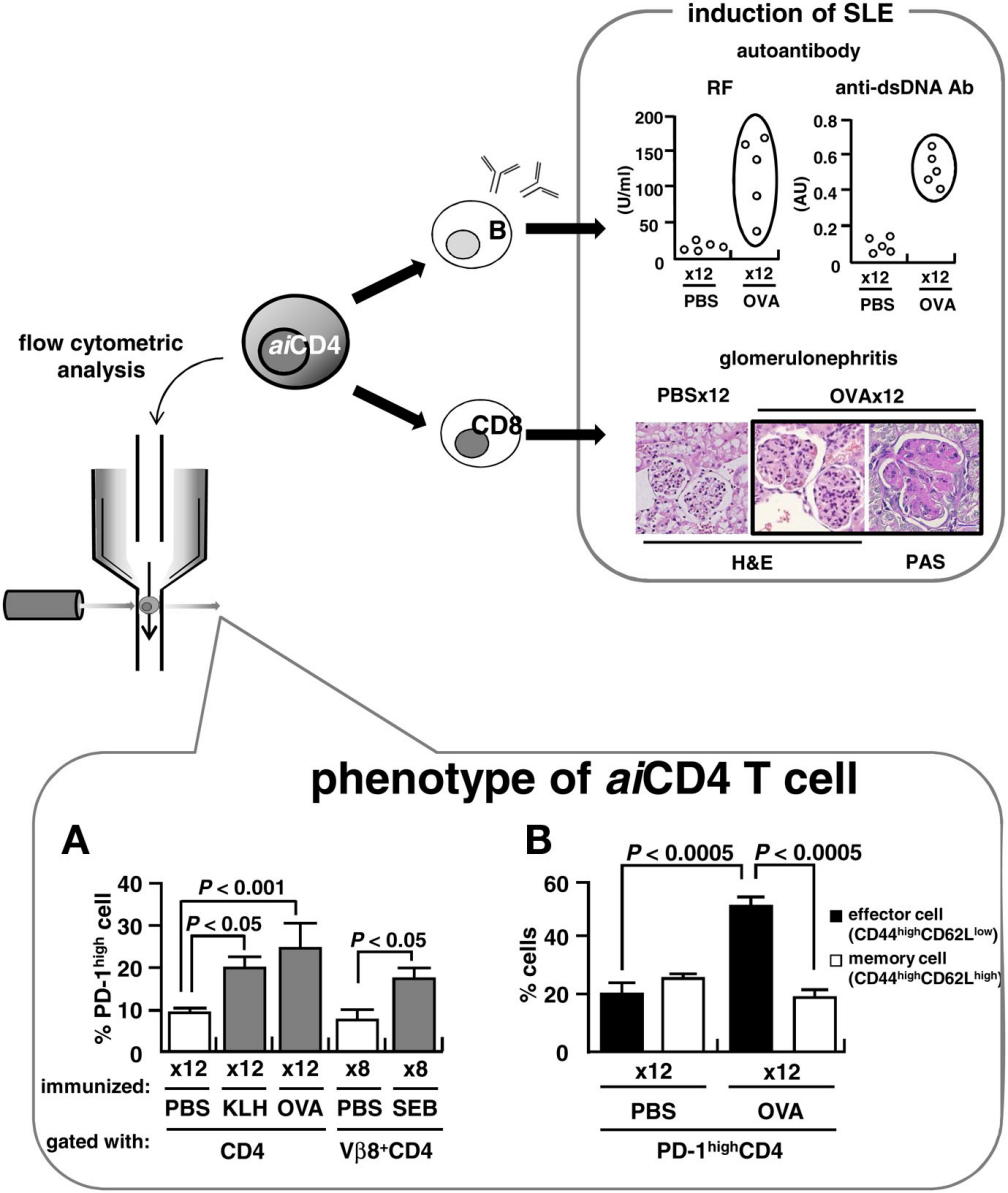
**Pathogenesis of SLE and *ai*CD4 T cell**. BALB/c mice were repeatedly injected i.p. with 100 μg of keyhole limpet hemocyanin (KLH), 500 μg of OVA, 25 μg of staphylococcal enterotoxin B (SEB), or PBS every 5 days. Adjuvants were not used. **(A,B)** Flow cytometry analyses of CD4 T cells following 12× repeated immunization with antigens (lower). Cells were stained on ice in the dark for 30 min with PerCP Cy5.5-conjugated anti-CD4 antibody (RM4-5), APC-conjugated anti-CD62L antibody (MEL-14), PE-conjugated anti-PD-1 antibody (J43) and anti-Vβ8 TCR antibody (F23.1), or FITC-conjugated anti-CD44 antibody (IM7). Samples were analyzed using a BD PharMingen FACSCalibur instrument. Effector and memory functions of PD-1^high^ CD4 T cells were determined by expression of CD44^high^CD62L^low^ and CD44^high^CD62L^high^ markers, respectively.

To further characterize the surface marker of *ai*CD4 T cell, we generated *ai*CD4 T cells in BALB/c mice, a strain that is normally not prone to autoimmune disease: 8-week-old BALB/c mice were repeatedly immunized (12×) with 100 μg KLH (Sigma, St. Louis, MO, USA), 500 μg OVA (grade V; Sigma), 25 μg SEB (Toxin Technologies, Sarasota, FL, USA), or PBS by means of i.p. injection every 5 days. This protocol has been previously found to generate *ai*CD4 T cells capable of inducing a variety of autoantibodies and pathological lesions identical to those seen in SLE (Tsumiyama et al., 2009). We found a significant expansion of CD4 T cells that expressed the PD-1^high^ marker (Figure 1A). These PD-1^high^ CD4 T cells further expressed CD44^high^CD62L^low^ effector T cell markers, but not CD44^high^CD62L^high^ memory T cell markers (Figure 1B). In addition, these cells expressed CD27^low^, CD45RB^low^, and CD122 ^high^markers (Miyazaki et al., 2013). We found that transfer of these PD-1-positive effector CD4 T cells into naïve recipients induced the generation of rheumatoid factor (RF) and anti-dsDNA antibodies. RF levels in the recipients were 39.36 ± 10.92 U/ml (*n* = 3) vs. 11.38 ± 5.21 U/ml (*n* = 7) in the controls (*P* < 0.0005), while anti-dsDNA antibody was 0.43 ± 0.02 AU (*n* = 3) vs. 0.25 ± 0.03 AU (*n* = 7) (*P* < 0.01), respectively.

Upon encounter with antigen, naïve CD4 T cells normally mature into CD27^low^, CD127^low^, CCR7^low^, CD44^high^CD62L^low^ effector cells (McKinstry et al., 2007; Wang et al., 2007). These effector cells subsequently differentiate into memory cells accompanied by increased expression of CD27, CD62L, CD127, and finally of PD-1 (Duraiswamy et al., 2011).

PD-1 belongs to the CD28 superfamily and is expressed on regulatory T cells (Treg) (Francisco et al., 2009), T follicular helper (Tfh) cells (Haynes et al., 2007), memory T cells, and exhausted CD8 T cells (Duraiswamy et al., 2011; Jin et al., 2011). PD-1 conveys a negative signal that causes reduced production of T cell cytokines, including IFNγ, TNFα, and IL-2 (Riella et al., 2012), and induces T cell tolerance (Jin et al., 2011; Riella et al., 2012).

We, however, saw no increase in FoxP3^+^CD25^+^ Treg upon repeated immunization with antigen in BALB/c mice. While CD25^-^CD4 T cells were increased in OVA-immunized mice as compared to PBS-treated mice; 80.1 vs. 68.3% (*P* < 0.01), the signal ratio of gene expression under microarray analyses of the CD4 T cell of the mice immunized 12× with OVA relative to PBS-treated mice was 0.98 for FoxP3, 1.11 for Erg-2, and 1.29 for LAG3, and thus, the finding indicates that *ai*CD4 T cell is distinct from regulatory CD4 T subsets. Further, while Tfh markers, B cell leukemia/lymphoma 6 (Bcl6), inducible T cell co-stimulator (ICOS), and chemokine (C-X-C motif) receptor 5 (CXCR5), were also not significantly increased under microarray analyses, further studies should be required concerning the relationship between *ai*CD4 T cell and Tfh or exhaust T cell.

The cells that are expanded upon repeated immunization with antigen are the effector CD4 T cells uniquely expressing memory PD-1 marker. This effector CD4 T cell population would be unique in that despite expression of the PD-1 marker it shows increased production of IL-2 and IL-6 (Miyazaki et al., 2013). Production of IL-2 should normally be suppressed when PD-1 is expressed (Latchman et al., 2001; Riella et al., 2012), however, as shown by several studies, sufficiently strong signaling through CD28 and/or IL-2 receptor can overcome PD-1 inhibitory signaling (Nurieva et al., 2006; Bertsias et al., 2009). In fact, the PD-1-expressing CD4 T cell population which we found was significantly increased both in NZB/W F1 mice (Kasagi et al., 2010) and in the peripheral blood of patients with SLE (Liu et al., 2009). Since this CD4 T cells induce varieties of autoantibodies and pathological lesions identical to those seen in SLE upon transfer into naïve recipients (Tsumiyama et al., 2009), it appears that this PD-1-expressing effector CD4 T cell is an activated T cell type which has overcome PD-1 inhibition.

## Role of B Cell: Is T Cell-Centered Disease Mechanism Valid?

While B cells produce autoantibodies, and intrinsic biochemical abnormalities in B cell, and T cell as well, can induce SLE, studies show that autoantibodies are produced from B cells that have undergone somatic hypermutation (SHM) with affinity maturation and class switching (Weinstein et al., 2012). Autoantibodies arise predominantly from non-autoreactive B cells that diversify immunoglobulin genes *via* SHM: SHM normally diversifies antibody genes during physiological responses to foreign immunogen within the microenvironment of the germinal center and thus, autoantibody-producing B cells are generated *de novo* at periphery (Guo et al., 2010; Weinstein et al., 2012). Therefore, T cell-centered disease mechanism seems valid in the pathogenesis of systemic autoimmunity.

## References

[B1] Bertsias G. K. Nakou M. Choulaki C. Raptopoulou A. Papadimitraki E. Goulielmos G. (2009). Genetic, immunologic, and immunohistochemical analysis of the programmed death 1/programmed death ligand 1 pathway in human systemic lupus erythematous. Arthritis Rheum. 60, 207–218 10.1002/art.24227 19116915

[B2] Duraiswamy J. Ibegbu C. C. Masopust D. Miller J. D. Araki K. Doho G. H. (2011). Phenotype, function, and gene expression profiles of programmed death-1^hi^ CD8 T cells in healthy human adults. J. Immunol. 186, 4200–4212 10.4049/jimmunol.1001783 21383243 PMC3723805

[B3] Francisco L. M. Salinas V. H. Brown K. E. Vanguri V. K. Freeman G. J. Kuchroo V. K. (2009). PD-L1 regulates the development, maintenance, and function of induced regulatory T cells. J. Exp. Med. 206, 3015–3029 10.1084/jem.20090847 20008522 PMC2806460

[B4] Fu S. M. Deshmukh U. S. Gaskin F. (2011). Pathogenesis of systemic lupus erythematosus revisited 2011: end organ resistance to damage, autoantibody initiation and diversification, and HLA-DR. J. Autoimmun. 37, 104–112 10.1016/j.jaut.2011.05.004 21632208 PMC3173577

[B5] Guo W. Smith D. Aviszus K. Detanico T. Heiser R. A. Wysocki L. J. (2010). Somatic hypermutation as a generator of antinuclear antibodies in a murine model of systemic autoimmunity. J. Exp. Med. 207, 2225–2237 10.1084/jem.20092712 20805563 PMC2947070

[B6] Haynes N. M. Allen C. D. Lesley R. Ansel K. M. Killeen N. Cyster J. G. (2007). Role of CXCR5 and CCR7 in follicular Th cell positioning and appearance of a programmed cell death gene-1high germinal center-associated subpopulation. J. Immunol. 179, 5099–5108 17911595 10.4049/jimmunol.179.8.5099

[B7] Jin H. T. Ahmed R. Okazaki T. (2011). Role of PD-1 in regulating T-cell immunity. Curr. Top. Microbiol. Immunol. 350, 17–37 10.1007/82_2010_116 21061197

[B8] Kasagi S. Kawano S. Okazaki T. Honjo T. Morinobu A. Hatachi S. (2010). Anti-programmed cell death 1 antibody reduced CD4^+^PD-1^+^ T cells and relieves the lupus-like nephritis of NZB/W F1 mice. J. Immunol. 184, 2337–2347 10.4049/jimmunol.0901652 20139271

[B9] Latchman Y. Wood C. R. Chernova T. Chaudhary D. Borde M. Chernova I. (2001). PD-L2 is a second ligand for PD-1 and inhibits T cell activation. Nat. Immunol. 2, 261–268 10.1038/85330 11224527

[B10] Liu M. F. Weng C. T. Weng M. Y. (2009). Variable increased expression of program death-1 and program death-1 ligands on peripheral mononuclear cells is not impaired in patients with systemic lupus erythematosus. J. Biomed. Biotechnol. 2009, 406136 10.1155/2009/729197 19759858 PMC2744882

[B11] McKinstry K. K. Golech S. Lee W. H. Huston G. Weng N. P. Swain S. L. (2007). Rapid default transition of CD4 T cell effectors to functional memory cells. J. Exp. Med. 204, 2199–2211 10.1084/jem.20070041 17724126 PMC2118696

[B12] Miyazaki Y. Tsumiyama K. Yamane T. Ito M. Shiozawa S. (2013). Expansion of PD-1-positive effector CD4 T cells in an experimental model of SLE: contribution to the self-organized criticality theory. Kobe J. Med. Sci. [Epub ahead of print]. 23756664

[B13] Nurieva R. Thomas S. Nguyen T. Martin-Orozco N. Wang Y. Kaja M. K. (2006). T-cell tolerance or function is determined by combinatorial costimulatory signals. EMBO J. 25, 2623–2633 10.1038/sj.emboj.7601146 16724117 PMC1478197

[B14] Perry D. Sang A. Yin Y. Zheng Y. Y. Morel L. (2011). Murine models of systemic lupus erythematosus. J. Biomed. Biotechnol. 2011, 271694 10.1155/2011/271694 21403825 PMC3042628

[B15] Riella L. V. Paterson A. M. Sharpe A. H. Chandraker A. (2012). Role of the PD-1 pathway in the immune response. Am. J. Transplant. 12, 2575–2587 10.1111/j.1600-6143.2012.04224.x 22900886 PMC3784243

[B16] Shiozawa S. (2011). Cause of systemic lupus erythematosus: a novel self-organized criticality theory of autoimmunity. Exp. Rev. Clin. Immunol. 7, 715–717 10.1586/eci.11.54 22014010

[B17] Shiozawa S. (2012). Pathogenesis of SLE and *ai*CD4 T cell: new insight on autoimmunity. Joint Bone Spine 79, 428–430 10.1016/j.jbspin.2012.03.010 22717290

[B18] Tsumiyama K. Miyazaki Y. Shiozawa S. (2009). Self-organized criticality theory of autoimmunity. PLoS ONE 4:e8382 10.1371/journal.pone.0008382 20046868 PMC2795160

[B19] Wang Y. Zhang H. X. Sun Y. P. Liu Z. X. Liu X. S. Wang L. (2007). Rig-I^−/−^ mice develop colitis associated with down regulation of Gαi2. Cell Res. 17, 858–868 10.1038/cr.2007.81 17893708

[B20] Weinstein J. S. Hernandez S. G. Craft J. (2012). T cells that promote B-Cell maturation in systemic autoimmunity. Immunol. Rev. 247, 160–171 10.1111/j.1600-065X.2012.01122.x 22500839 PMC3334351

